# Resistance of Human Liver Mesenchymal Stem Cells to FAS-Induced Cell Death

**DOI:** 10.3390/cimb44080236

**Published:** 2022-07-30

**Authors:** Irina V. Kholodenko, Alisa M. Gisina, Garik V. Manukyan, Alexander G. Majouga, Elena V. Svirshchevskaya, Roman V. Kholodenko, Konstantin N. Yarygin

**Affiliations:** 1 Orekhovich Institute of Biomedical Chemistry, 119121 Moscow, Russia; alisa.gisina@gmail.com (A.M.G.); kyarygin@yandex.ru (K.N.Y.); 2 Petrovsky Russian Research Center of Surgery, 119991 Moscow, Russia; drmanukyan@mail.ru; 3 Faculty of Chemical and Pharmaceutical Technologies and Biomedical Products, Mendeleev University of Chemical Technology of Russia, 125047 Moscow, Russia; alexander.majouga@gmail.com; 4 Shemyakin-Ovchinnikov Institute of Bioorganic Chemistry, Russian Academy of Sciences, 117997 Moscow, Russia; esvir@mail.ibch.ru (E.V.S.); khol@mail.ru (R.V.K.)

**Keywords:** human liver mesenchymal stem cells, Fas/CD95, FasL, anti-Fas mAb, ATP, mitochondrial membrane potential, cell death, apoptosis resistance

## Abstract

Mesenchymal stem cells (MSCs) have a pronounced therapeutic potential in various pathological conditions. Though therapeutic effects of MSC transplantation have been studied for a long time, the underlying mechanisms are still not clear. It has been shown that transplanted MSCs are rapidly eliminated, presumably by apoptosis. As the mechanisms of MSC apoptosis are not fully understood, in the present work we analyzed MSC sensitivity to Fas-induced apoptosis using MSCs isolated from the biopsies of liver fibrosis patients (L-MSCs). The level of cell death was analyzed by flow cytometry in the propidium iodide test. The luminescent ATP assay was used to measure cellular ATP levels; and the mitochondrial membrane potential was assessed using the potential-dependent dye JC-1. We found that human L-MSCs were resistant to Fas-induced cell death over a wide range of FasL and anti-Fas mAb concentrations. At the same time, intrinsic death signal inducers CoCl_2_ and staurosporine caused apoptosis of L-MSCs in a dose-dependent manner. Despite the absence of Fas-induced cell death treatment of L-MSCs with low concentrations of FasL or anti-Fas mAb resulted in a cellular ATP level decrease, while high concentrations of the inducers caused a decline of the mitochondrial membrane potential. Pre-incubation of L-MSCs with the pro-inflammatory cytokine TNF-α did not promote L-MSC cell death. Our data indicate that human L-MSCs have increased resistance to receptor-mediated cell death even under inflammatory conditions.

## 1. Introduction

Multiple experimental works and clinical trials have shown that mesenchymal stem cells (MSCs) have a pronounced therapeutic potential in various pathological conditions [1,2,3]. Among the main mechanisms of the therapeutic action of MSCs are the immunomodulatory properties of MSCs mediated by the secretion of a wide range of humoral factors and extracellular vesicles that promote tissue regeneration, angiogenesis, and reduce inflammatory reactions [4]. Despite the fact that the therapeutic mechanisms of MSCs have been studied for quite a long time, a common opinion on this matter has not yet been formed. Over the past few years, it has been proven that transplanted MSCs remain viable in the recipient’s body for a very short time [5,6]. Several studies have shown that after intravenous MSC transplantation only apoptotic cells and apoptotic bodies are found in the lungs and liver within 4–12 h [7,8]. In addition, the immunomodulatory and anti-inflammatory function of apoptotic bodies derived from MSCs was shown in various animal models of inflammatory and autoimmune diseases [9,10,11]. Apoptotic MSCs had a hepatoprotective effect in the model of CCl_4_-induced liver injury [12]. They also enhanced angiogenesis and improved functional recovery in animals with myocardial infarction due to the regulation of autophagy in the recipient endothelial cells [13]. Thus, the concept of another potential mechanism of the MSC therapeutic action through the formation of apoptotic bodies has been formed by now. Despite many studies demonstrating the immunomodulatory properties and therapeutic efficacy of apoptotic MSCs, the triggering of the cell death signal and the mechanism of apoptosis in these cells are poorly understood. However, the Fas–FasL interaction is considered one of the main causes of apoptosis in transplanted MSCs [14,15].

Fas (CD95) is a membrane receptor that belongs to the death receptor superfamily. The CD95 ligand (FasL) belongs to the family of death ligands along with TNF-α and TRAIL. The interaction of FasL with the Fas/CD95 receptor triggers a cascade of signals leading to apoptotic cell death. FasL is expressed either as a membrane-bound protein or in a soluble form [16]. Fas/CD95 was originally identified in the immune cells as an apoptosis-inducing receptor [17]. Later, its role as a regulator of various non-apoptotic functions was identified in a wide spectrum of cell types. For example, it has been shown that Fas/CD95 promotes tumor growth and invasion [18,19,20], induces neurogenesis [21,22], and enhances the proliferation of T-lymphocytes [23] and fibroblasts [24]. In a number of studies, it was shown that, along with the proapoptotic effect [25], FasL induces MSC proliferation and their phenotype switching to a more stem state [26], as well as suppresses adipogenic differentiation [27]. It is noteworthy that the mode of FasL activity may depend on its concentration [26,27].

As repeatedly reported, MSCs can be isolated from various tissue sources and have stable specific characteristics including adhesion to culture plastic, expression of mesenchymal markers CD44, CD90, CD105, and the ability to differentiate into mesodermal lineages. MSC-like cells can also be obtained from the adult human liver [28]. In addition to the above properties, liver MSCs (L-MSCs) have an improved ability to differentiate into hepatocyte-like cells compared to the umbilical cord-derived MSCs [29], a feature that can be important for liver regeneration. L-MSCs demonstrate more pronounced immunomodulatory and decreased pro-angiogenic properties compared to adipose tissue-derived MSCs [30]. L-MSCs have shown marked therapeutic effects in various animal models of liver [31], kidney [32], and pancreas [33] pathologies.

In the present work, the responses of human L-MSCs to various cell death inducers, such as FasL, anti-Fas mAb, staurosporine (STS), and cobalt (II) chloride (CoCl_2_) were studied. We have shown for the first time that human L-MSCs are resistant to Fas-induced cell death. Neither FasL nor anti-Fas mAb induced cell death in human L-MSCs over a wide range of concentrations. Nevertheless, there was a marked decrease in the level of intracellular ATP after L-MSCs incubation with low doses of FasL or anti-Fas mAb, and mitochondrial depolarization after L-MSC treatment with high doses of FasL or anti-Fas mAb. Pretreatment of the human L-MSCs with the pro-inflammatory cytokine TNF-α did not affect Fas-induced apoptosis resistance of L-MSCs. At the same time, the intrinsic death signal inducers CoCl_2_ and STS triggered L-MSC cell death in a dose-dependent manner.

## 2. Materials and Methods

### 2.1. Isolation and Cultivation of Human Liver MSCs

Liver biopsies were provided by the Department of Emergency Surgery and Portal Hypertension of the Petrovsky Russian Research Center of Surgery. The study was approved and supervised by the Institutional Ethics Committee (Approval Protocol No. 4, 21 April 2022). Written informed consent was obtained from all patients. Biological samples were handled using anonymous codes in accordance with the Federal Law on Personal Data (No. 152-FZ, 27 July 2006). L-MSCs were isolated from the biopsy material obtained from three patients with liver fibrosis, according to the method described previously [34]. Briefly, the liver tissue samples were minced with a scalpel. Then, the minced tissue was placed in a solution of type IV collagenase (0.1% *w*/*v*) (Gibco, Waltham, MA USA) and incubated for 30 min in a CO_2_ incubator. Processed liver tissue was washed twice in PBS (PanEco, Moscow, Russia) supplemented with 1% FBS (Gibco, Waltham, MA USA). After that, the complete growth medium was added, and the suspension was maintained in culture flasks (75 cm^2^) until adherent cells appeared. The MSC cultivation was carried out in a complete growth medium DMEM/F12 containing 10% FBS, 100 U/mL penicillin, and 100 μg/mL streptomycin (all of them Gibco, Waltham, MA USA). The cultivation was carried out under standard conditions in a CO_2_ incubator (37 °C, 5% CO_2_, 80% humidity). Upon reaching 80–90% confluency, the cells were passaged. In all experiments, the cells from no later than the 5th passage were used.

### 2.2. Flow Cytometry Analysis of the MSC Markers and CD95

The cells were trypsinized and washed twice in PBS (PanEco, Moscow, Russia) supplemented with 1% FBS (Gibco, Waltham, MA USA) by centrifugation for 5 min at 300× *g*. The cells were incubated with anti-CD14-FITC, anti-CD34-APC, anti-CD45-PE, anti-CD29-APC, anti-CD44-FITC, anti-CD73-PE, anti-CD105-PE, anti-CD95-APC (BD Biosciences, Franklin Lakes, NJ, USA) at concentrations recommended by the manufacturer, for 1 h at 4 °C in the dark. At the end of the incubation, the cells were washed twice in PBS by centrifugation. Fluorescence intensity was analyzed using BD FACSAria III flow cytometer (BD Biosciences, Franklin Lakes, NJ, USA). At least 10^4^ events were recorded in each sample. The results were processed using the FlowJo_V10 (FlowJo™, Ashland, OR, USA). The relative fluorescence intensity (RFI) of marker expression was calculated as the ratio of the specific fluorescence of cell staining with fluorescently labeled antibodies and the autofluorescence of control unstained cells.

### 2.3. Cell Death Analysis

Cell death was assessed by flow cytometry in the propidium iodide (PI) test, as described previously [35]. Staurosporine (STS) (Merck Life Science LLC, Moscow, Russia) at concentrations of 0.2–1 µM, CoCl_2_ (Merck Life Science LLC, Moscow, Russia) at concentrations of 100–400 µM, recombinant human Fas ligand (FasL, TNFSF6; BioLegend, San Diego, CA, USA) at concentrations of 50–500 ng/mL and anti-Fas mAb (Clone DX2; BioLegend, San Diego, CA, USA) at concentrations of 0.05–2 µg/mL were used as the cell death inducers. The cells were preliminarily plated in the wells of a 12-well plate (5 × 10^5^ cells/well) until adhesion, after which the inducers were added and L-MSCs were incubated for 72 h.

In order to analyze the effect of pro-inflammatory cytokines on the sensitivity of L-MSCs to Fas-induced cell death, the cells were preincubated with TNF-α (20 ng/mL) (Merck Life Science LLC, Moscow, Russia) for 48 h and then treated with various concentrations of FasL or anti-Fas mAb (BioLegend, San Diego, CA, USA) for another 24 h. After incubation, the cells were trypsinized and washed twice in PBS by centrifugation. The cell pellet was resuspended in ice-cold 70% ethanol for 1 h to fix and permeabilize the cells. Then the cells were washed twice in PBS, resuspended in 100 µL PBS-based staining solution supplemented with PI (10 µg/mL, Merck Life Science LLC, Moscow, Russia) and RNase (10 µg/mL, Merck Life Science LLC, Moscow, Russia) and incubated for 15 min. After this, the volume of the suspension was adjusted to 500 µL with PBS, and fluorescence intensity was analyzed on a BD FACSAria III flow cytometer-sorter (BD Biosciences, Franklin Lakes, NJ, USA). At least 10^4^ events were recorded in each sample. The results were processed using the FlowJo_V10 (FlowJo™, Ashland, OR, USA).

A colorimetric MTT test was used to assess the viability of human L-MSCs after treatment with FasL, anti-Fas mAb, CoCl_2_, or STS (see Supplementary Files).

### 2.4. ATP Level in the L-MSCs

ATP levels in the L-MSC cultures were determined by the Luminescent ATP Detection Assay Kit (Abcam, Waltham, MA, USA) according to the manufacturer’s instructions. The L-MSCs (10^4^ cells/well) were seeded at 48 h beforehand on a 96-well plate. After that, cell death inducers were added and incubated for 4 h. At the end of incubation, 50 μL of the detergent was added to each well and incubated for 5 min on an orbital shaker at 600–700 rpm. Then, 50 µL of the substrate was added to the wells and incubated for 5 min on an orbital shaker and for an additional 10 min in the dark. Wells with complete growth medium without cells were used as a negative control. Each point in one experiment was run in triplicate. Luminescence was measured on a Tecan infinite M200 Pro plate reader (Tecan, Männedorf, Switzerland). Data are presented as control-normalized luminescent relative light unit (RLU) values.

### 2.5. Assessment of the Mitochondrial Membrane Potential

Changes in the level of the mitochondrial membrane potential were analyzed by flow cytometry using the potential-dependent dye JC-1 (Merck Life Science LLC, Moscow, Russia), as described previously [36]. The L-MSCs plated in 12-well plates at 5 × 10^5^ cells/well were incubated for 48 h. Cell death inducers were added to the cells at various concentrations for 4 h. After that, the cells were trypsinized, washed twice in complete growth medium, and incubated for 15 min at room temperature in complete growth medium supplemented with 2 μg/mL JC-1. The cells were then washed twice in ice-cold PBS. Fluorescence intensity was analyzed using a BD FACSAria III flow cytometer sorter (BD Biosciences, Franklin Lakes, NJ, USA). At least 10^4^ events were recorded in each sample. The results were processed using the FlowJo_V10 (FlowJo™, Ashland, OR, USA).

### 2.6. Statistical Analysis

Statistical data processing was performed using the SigmaPlot 12.5 soft (Systat Software Inc., Palo Alto, CA, USA). Data are presented as the mean ± SEM. All measurements were performed in at least three replicates. Statistical differences between groups were calculated by Student’s *t*-test; *p* < 0.05 was regarded as significant.

## 3. Results

### 3.1. Phenotypic Characteristics of L-MSCs

**Figure 1:**
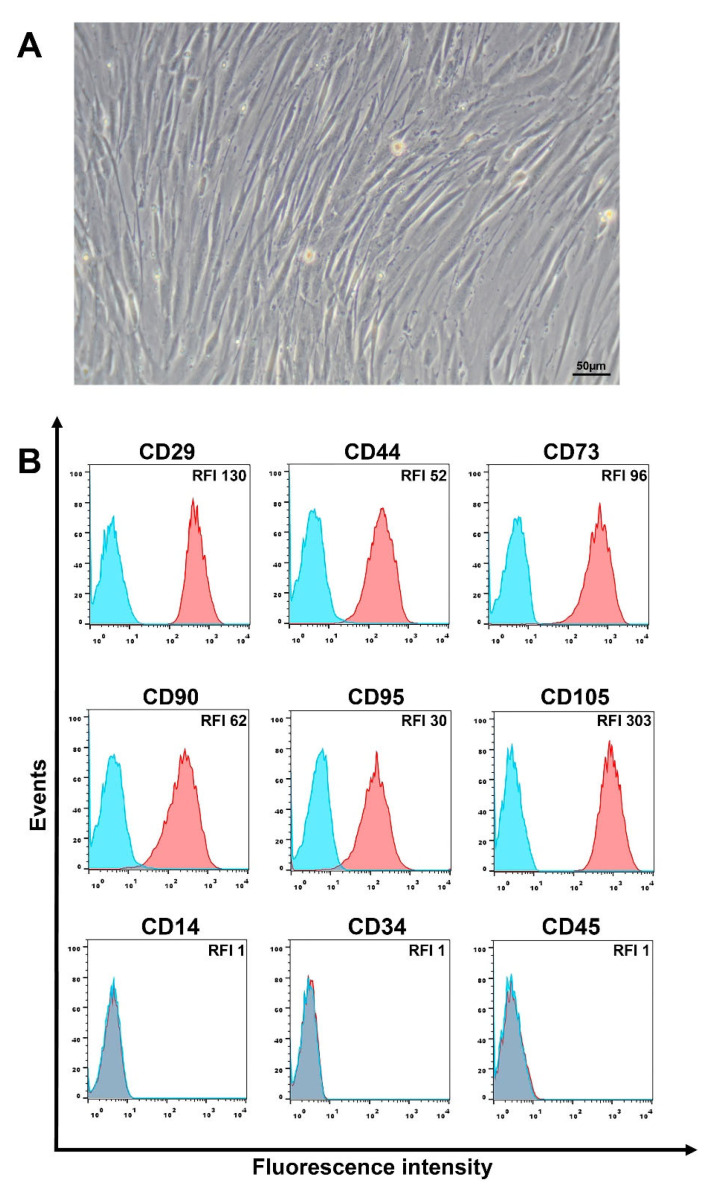
Morphology and phenotype of L-MSCs. (**A**) Phase-contrast microscopy of L-MSC culture. Cell cultures were photographed using the Axiovert 40 CFL (Carl Zeiss, Oberkochen, Germany) inverted microscope and the Nikon D5000 digital camera (Nikon, Tokyo, Japan). (**B**) Expression of MSC markers, CD95, and immune cell markers on the surface of L-MSCs. The blue peaks correspond to the control unstained cells; the red peaks to the cells stained with the corresponding antibodies. Representative data for one culture of L-MSCs are presented. RFI—relative fluorescence intensity.

Human L-MSCs obtained from the liver biopsies of liver fibrosis patients have a fibroblast-like morphology (Figure 1A) and are able to differentiate into mesodermal lineages (Figure S1). Phenotypically, these cells have high homogeneous expression of mesenchymal stem/stromal cell markers, such as CD29, CD44, CD73, CD90, and CD105. In addition, L-MSCs are also negative for the expression of immune cell markers (Figure 1B). Similar results of the phenotypic analysis were obtained for MSCs isolated from the biopsy specimens from all three patients.

Analysis of CD95 expression in three cultures of L-MSCs showed that all cell populations express it at a high level (Figure 1B). Probably, this high and homogeneous Fas/CD95 expression may indicate the potential sensitivity of the cells to Fas-induced cell death due to the interaction of CD95 on the cell surface with FasL or anti-Fas mAb.

### 3.2. Liver MSCs Are Resistant to Fas-Induced Cell Death

**Figure 2:**
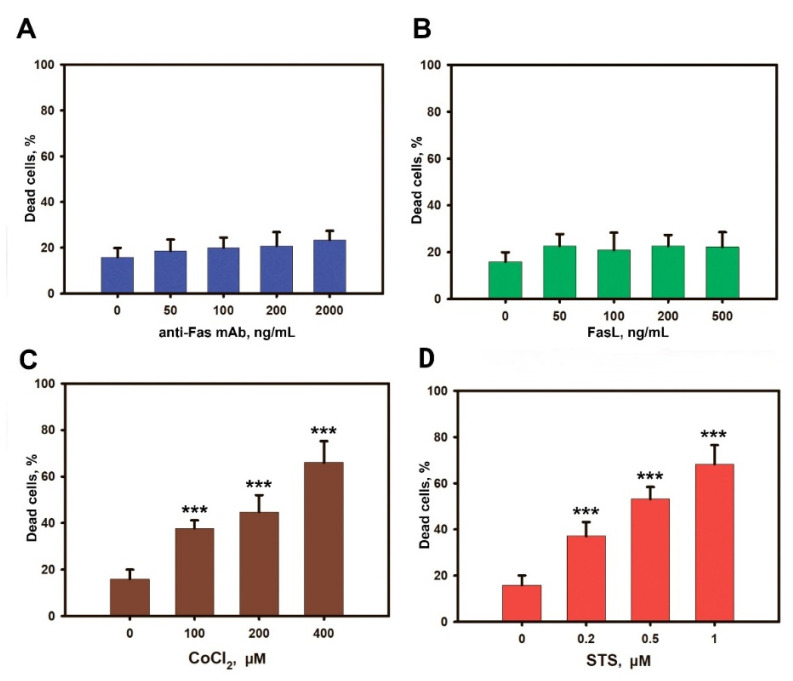
Flow cytometry analysis of L-MSC cell death. L-MSCs were incubated with anti-Fas mAb (**A**); FasL (**B**); CoCl_2_ (**C**), or STS (**D**) for 72 h. Data are presented as the percentages of the cells with the fragmented DNA. The results from three L-MSC cultures isolated from individual patients are shown. *** *p* < 0.001.

L-MSCs were incubated with FasL or anti-Fas mAb for 72 h and the percentages of the dead cells were evaluated by flow cytometry. In addition to receptor-dependent apoptosis (extrinsic pathway) induced through CD95, we also analyzed the intrinsic pathway of cell death induced by STS and CoCl_2_ (Figure 2). Neither anti-Fas mAb nor FasL induced cell death in L-MSCs in the selected concentration range (*p* > 0.001) (Figure 2A,B). L-MSC cell death was also studied after treatment with these inducers for 24 h and 48 h, and no apoptosis-inducing effect was found (Figures S2–S4). The viability of L-MSCs was assessed in the MTT test after cell treatment with FasL or anti-Fas mAb. Both inducers did not affect the viability of L-MSCs in the selected concentration range (Figure S5A,B). As a positive control for the metabolic activity recorded by the MTT assay, L-MSCs were incubated with various dilutions of STS and CoCl_2_. STS and CoCl_2_ both induced decrease in the L-MSCs viability in a dose-dependent manner (Figure S5C,D).

The obtained results may indicate the resistance of human L-MSCs to receptor-mediated cell death via Fas/CD95 ligation. As controls, we also have used two known death inducers: CoCl_2_ and STS. We have shown a dose-dependent increase in the percentages of dead L-MSCs after treatment with both inducers (Figure 2C,D; *p* < 0.001).

### 3.3. High Doses of Anti-Fas mAb or FasL Induce Changes in Mitochondrial Membrane Potential in Liver MSCs

An important feature of the early stages of the apoptotic cascade is the disruption of the normal functioning of mitochondria, including changes in the membrane potential. Potential-dependent dyes, which freely penetrate the cell plasma membrane, are routinely used to monitor the state of the mitochondria. The JC-1 dye has the property of potential-dependent accumulation in mitochondria. At high mitochondria membrane potential, JC-1 dye monomer forms red fluorescent “J-aggregates” with an emission maximum at ~590 nm (FL2 channel) accumulated within the mitochondria. At a low membrane potential, the JC-1 dye is present as monomers, devoid of the FL2 channel fluorescence and preserving it in the FL1 channel (529 nm). Therefore, a decrease in the intensity ratio of red/green fluorescence indicates depolarization of mitochondria (FL2/FL1) [37]. Using the JC-1 dye, we analyzed the changes that occur in the mitochondria of three L-MSC cultures after 4 h incubation with the cell death inducers. Treatment of L-MSCs with low concentrations of anti-Fas mAb or FasL (50 and 100 ng/mL) did not change the mitochondrial membrane potential (Figure S6).

**Figure 3:**
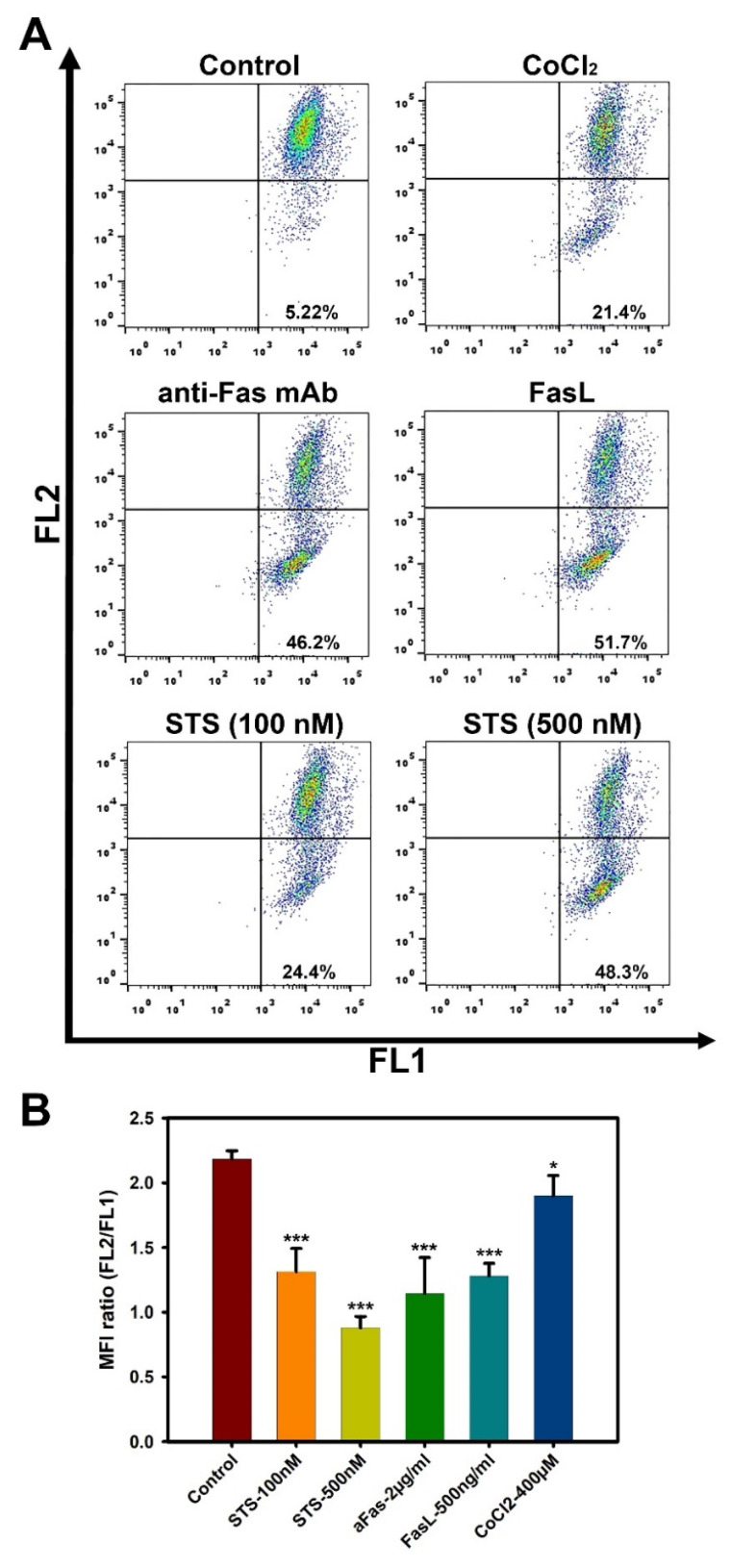
Flow cytometry analysis of mitochondrial membrane potential using JC-1 dye. L-MSCs were incubated with anti-Fas mAb (2 µg/mL), FasL (500 ng/mL), CoCl_2_ (400 µM), or STS (100 nM and 500 nM) for 4 h. (**A**) Two-parameter histograms of fluorescence intensity distribution in FL1 and FL2 channels. Representative data obtained from a single culture of L-MSCs. (**B**) MFI ratios FL2/FL1. Averaged data from three patient-derived L-MSC cultures. * *p* < 0.05, *** *p* < 0.001.

Despite the fact that anti-Fas mAb or FasL did not induce cell death in L-MSCs in the concentration range studied (Figure 2A,B), short-term incubation (4 h) of the cells with these inducers at high doses (2 μg/mL anti-Fas mAb or 500 ng/mL FasL) resulted in a marked decrease in mitochondrial potential in a significant pool of cells (Figure 3).

It should be noted that only a part of the cells contained depolarized mitochondria after treatment with inducers. Table 1 presents data on the percentage of the cells with a reduced mitochondrial membrane potential in each of the three patient-derived cultures of L-MSCs.

Figure 3A and Table 1 demonstrate that about 50% of the cells in the total population of L-MSCs treated with high concentrations of anti-Fas mAb or FasL have a reduced mitochondrial membrane potential, indicating depolarization of mitochondria in these cells. The FL2/FL1 ratios in these samples decreased by almost 2 times compared to control cells (Figure 3B). We used two concentrations of STS (100 and 500 nM) in order to show the dose dependence of mitochondrial depolarization. Indeed, STS at 100 nM induced less depolarization than at 500 nM (Figure 3B). The percentages of L-MSCs with reduced mitochondrial membrane potentials were approximately 20% and 50% for 100 nM and 500 nM, respectively (Figure 3A, and Table 1). CoCl_2_ was less efficient in the induction of mitochondrial membrane potential in L-MSCs (Figure 3, and Table 1).

### 3.4. Low Concentrations of Anti-Fas mAbs or FasL Induce an ATP Level Decline in L-MSCs

Apoptosis is an ATP-dependent process. While early studies found a decrease in intracellular ATP mostly in necrotic cells [38], later an ATP level decline was also found at some stages of the apoptotic cascade [39,40]. We have analyzed the ATP level changes in the L-MSCs treated with the death inducers for 4 h.

**Figure 4:**
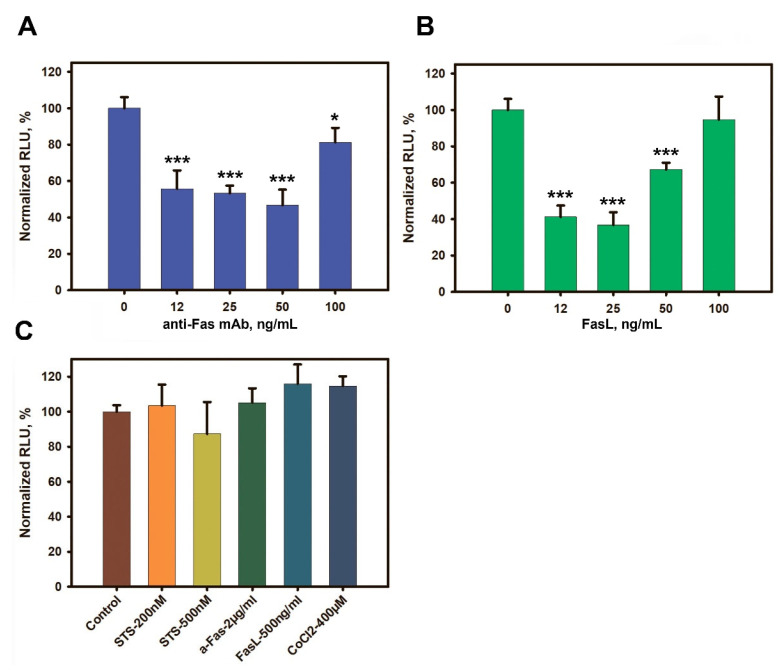
Effect of cell death inducers on the ATP levels in L-MSCs. L-MSCs were incubated with low concentrations of anti-Fas mAb (**A**), FasL (**B**) (0–100 ng/mL), and high concentrations of cell death inducers (**C**) for 4 h. The level of ATP is presented as relative light unit (RLU) values normalized to the control cells, where the luminescence of the control cells is taken as 100%. Averaged combined data from three patient-derived L-MSC cultures are shown. * *p* < 0.05, *** *p* < 0.001.

Low but not high doses of anti-Fas mAb or FasL caused a marked decline in ATP levels in the L-MSCs (Figure 4). Maximal effect of anti-Fas mAb was found at 12–50 ng/mL up to 40% RLU decrease compared to the control, while at 100 ng/mL it was only 20% (Figure 4A, *p* < 0.05). Close to it, FasL induced a decrease in ATP levels in 60% at 12–25 ng/mL and 30% at the concentration 50 ng/mL (*p* < 0.001). No effect was found at 100 ng/mL (Figure 4B, *p* > 0.05). High concentrations of anti-Fas mAb (2 µg/mL) or FasL (500 ng/mL), as well as STS and CoCl_2_, did not lead to the changes in ATP levels in L-MSCs after 4 h incubation (Figure 4C).

Despite that all three patient-derived L-MSC cultures were resistant to the induction of cell death through CD95, a number of changes, such as depolarization of mitochondria and decrease in ATP levels after their treatment with anti-Fas mAb or FasL, occurred, probably indicating the initiation of physiologically significant processes.

### 3.5. Pro-Inflammatory Cytokine TNF-α Does Not Increase the Sensitivity of L-MSCs to Fas-Induced Cell Death

For some cell types, pro-inflammatory cytokines such as IL-1β in combination with IFN-γ, as well as TNF-α have been shown to induce cytokine-dependent apoptosis [41,42]. These cytokines also increase the sensitivity of hepatocytes to Fas-induced cell death [43,44]. Moreover, TNF-α, along with TGFβ, are key pro-inflammatory cytokines involved in the development of liver fibrosis [45,46]. In preliminary experiments, we did not find any effect of the pro-inflammatory pro-fibrotic cytokines TNF-α and TGFβ, as well as the cytokines with anti-fibrotic properties, such as IFNγ [47] and IL6 [48], on the viability of L-MSCs (Figure S7). Taking into account the fact that TNFα plays a decisive role in the induction of apoptosis/autophagy of transplanted MSCs in various pathologies associated with the inflammatory process [49,50], we chose this cytokine for the potential sensitization of L-MSCs to Fas-induced apoptosis. Thus, we evaluated the effect of TNF-α on Fas-induced cell death in L-MSCs. Human L-MSCs were pretreated with TNF-α (20 ng/mL) for 48 h, and then incubated with anti-Fas mAb (2 μg/mL) or FasL (500 ng/mL) for 24 h. The level of cell death was assessed by the PI test.

**Figure 5:**
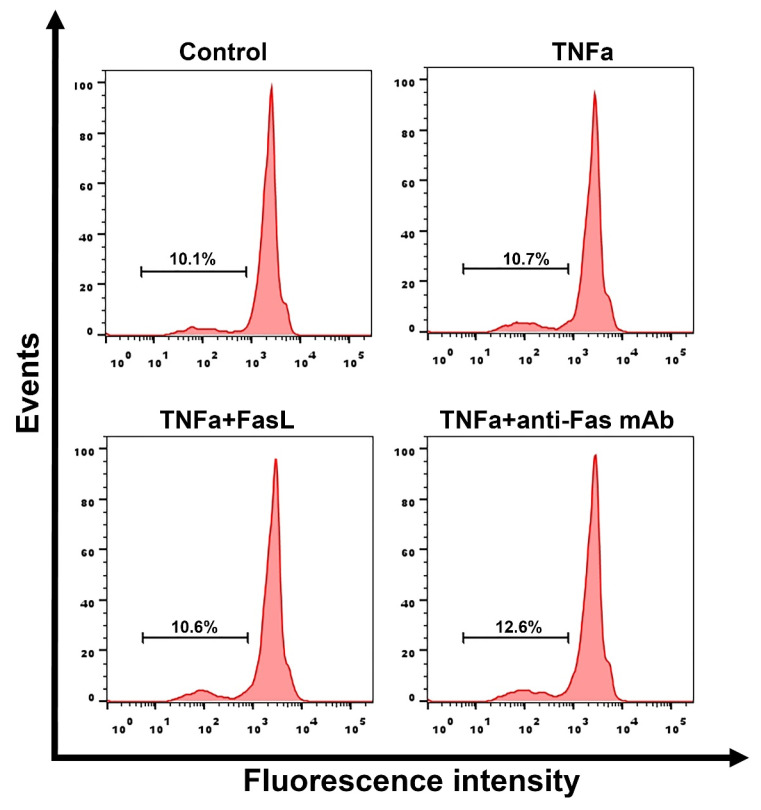
Cell death of L-MSCs pretreated with TNF-α. L-MSCs were incubated with 20 ng/mL TNF-α for 48 h and then incubated with anti-Fas mAb (2 µg/mL) or FasL (500 ng/mL) for 24 h. The bars indicate the percentage of cells with fragmented DNA. Representative data obtained for a single culture of L-MSCs are shown.

TNF-α alone at a concentration of 20 ng/mL did not induce cell death in any of the three patient-derived L-MSC cultures. Pretreatment of L-MSCs with TNF-α and anti-Fas mAb or FasL also did not result in cell death (Figure 5). These data may indicate that even under inflammatory conditions, human L-MSCs do not become sensitive to Fas-induced apoptosis.

## 4. Discussion

Liver progenitor cells, phenotypically and morphologically similar to mesenchymal stem/stromal cells, were first described by Herrera et al. in 2006 [51]. Later, several groups investigated these cells in the context of their functional characteristics, such as differentiation potential in vitro and in vivo, therapeutic potential in various models of liver diseases, and immunomodulatory properties [28]. In addition, L-MSCs have been studied as cell therapy for liver pathologies in clinical trials [52,53]. In this work, we have shown that human L-MSCs isolated from biopsy specimens from three liver fibrosis patients have a classic MSC phenotype, expressing characteristic markers. All cells in each of the three populations of L-MSCs expressed CD29, CD44, CD73, CD90, and CD105, which is consistent with the results for the classic bone marrow-derived MSCs (BM-MSCs) phenotyping [54,55].

Most MSCs isolated from various tissue sources, including human L-MSCs, are characterized by high expression of the death receptor Fas/CD95 [56,57,58]. A number of studies have also shown that MSCs express and secrete FasL [59]. It has been shown that FasL expression partly mediates the immunomodulatory properties of MSCs [60,61,62]. It is expected that the high expression of Fas makes these cells potentially susceptible to Fas-induced apoptosis [14]. To date, there is no certain answer to the question of whether MSCs are resistant or susceptible to Fas-induced apoptosis, since the opposite results have been obtained in different studies. For example, fetal human MSCs respond to both STS (intrinsic mitochondrial apoptotic pathway) and FasL/anti-Fas mAb (extrinsic receptor-mediated apoptotic pathways) with a dose-dependent increase in apoptotic death [25,63], while adult BM-MSCs are resistant to receptor-mediated apoptotic pathways [59]. Moreover, BM-MSCs obtained from ankylosing spondylitis patients are more sensitive to receptor-mediated apoptosis than BM-MSCs obtained from healthy donors [64]. In addition, MSCs at late passages showed greater resistance to Fas-induced cell death compared to MSCs at early ones [26]. Probably, such differences can be associated with the individual characteristics of cells isolated from different sources.

In our work, we have shown for the first time that human L-MSCs are resistant to cell death induced both by FasL or anti-Fas mAb over a wide range of concentrations. These results are consistent with the previously published data, which showed that human undifferentiated BM-MSCs are resistant to receptor-induced cell death even at high concentrations of FasL or anti-Fas mAb [59,65]. Ham et al. [14] showed that FasL induces apoptosis only in MSCs pretreated with hydrogen peroxide. Initiation of BM-MSC differentiation in the osteogenic and/or adipogenic lineages made these cells sensitive to apoptotic stimuli [65]. In addition, Oliver et al. [65] demonstrated the resistance of undifferentiated BM-MSCs to STS, etoposide, and hypoxia. The authors showed that the blockade of apoptosis in undifferentiated BM-MSCs occurs at the mitochondrial level, since they did not detect the release of cytochrome C. In our work, we showed that CoCl_2_, which is a chemical inducer of hypoxia, and STS induced L-MSCs death in a dose-dependent manner. At the same time, we showed a rapid decrease in the mitochondrial potential for the selected working concentrations of CoCl_2_ and STS, which may indirectly indicate a release of cytochrome C and the normal passage of the intrinsic mitochondrial apoptotic pathway in these cells.

Much more ambiguous and interesting are the results obtained by inducing extrinsic receptor-mediated apoptotic pathways in MSCs. There are several studies showing that the induction of apoptosis in MSCs depends on the concentration of FasL [26,27]. Rippo et al. [27] showed that a low concentration of FasL (0.5 ng/mL) stimulated the proliferation of BM-MSCs, while 25 ng/mL induced a significant cell death stimulating more stem phenotype in the surviving cells. In addition, a high dose of FasL reversibly suppressed the adipogenic differentiation of BM-MSCs [27]. In another study [26], the doses of FasL up to 25 ng/mL stimulated adipose-derived MSCs proliferation, while the doses above 25 ng/mL predominantly induced apoptosis. The authors also found an increase in the “stemness” after the treatment with FasL at 50 ng/mL in the surviving cells, namely, a decrease in CD105 expression and an increase in CD73 one [26]. In our work, we demonstrated that the treatment of L-MSCs with low doses of FasL or anti-Fas mAb led to a marked decline of cellular ATP levels, while incubation of the cells with high doses of the inducers caused a decrease in mitochondrial membrane potential. Notably, these events occurred in the absence of cell death.

We suggest that the resistance of L-MSCs to Fas-induced cell death may be mediated by some specific mechanisms of resistance to apoptosis. It is known that the cell death receptors can induce both apoptosis and survival. Receptor-mediated apoptosis can be regulated at various levels, ranging from the death-inducing signaling complex (DISC) formation to mitochondrial outer membrane permeabilization (MOMP) and subsequent activation of effector caspases and nuclear fragmentation [66]. Therefore, the fate of the cells (death or survival) subjected to the stimulation through the death receptor can also be predetermined at different stages of the apoptotic cascade. For example, high constitutive expression of anti-apoptotic proteins and inhibitors of apoptosis such as c-FLIP [67], XIAP [68], or BCL-2 [69], can lead to the survival of the cells after the stimulation through the death receptors. In particular, resistance to Fas-induced apoptosis in human dermal fibroblasts, which are cells of mesenchymal origin, has been shown to be associated with the constitutive expression of c-FLIP. Inhibition of c-FLIP expression by cycloheximide or antisense FLIP oligonucleotides made these cells susceptible to extrinsic death receptor-mediated cell death [70]. High constitutive expression of the anti-apoptotic protein Bcl-XL protected human BM-MSCs from apoptosis [65].

Mitochondrial depolarization during cell death is associated with the MOMP pathway. MOMP was originally considered an irreversible stage and defined as an all-or-nothing event. However, it has recently been shown that even after MOMP, the mechanisms of cell death regulation can lead to cell survival [71]. Evidently, cell recovery after MOMP is not always possible. In the case of total mitochondria damage, the process of cell death is irreversible. Cell survival closely correlates with the maintenance of intact mitochondria that avoid MOMP, a condition referred to as incomplete MOMP [72]. These intact mitochondria serve as a critical pool for recovering the mitochondrial network, providing cell survival reviewed in [73]. For example, cell survival after MOMP may be promoted by the glycolytic enzyme glyceraldehyde-3-phosphate dehydrogenase (GAPDH) through its stable glycolytic role in ATP synthesis and the ability to transcriptionally stimulate autophagy to remove permeabilized non-functional mitochondria through mitophagy [74]. Mitophagy plays an important role in the resistance of MSCs to stressful stimuli leading to apoptosis. Due to mitophagy, the cell quickly gets rid of damaged functionally inactive mitochondria, maintaining intracellular homeostasis [75]. Most studies describe stem cells as being glycolytic [76,77]. In particular, it has been shown that anaerobic glycolytic metabolism predominates in human BM-MSCs, which maintains their stemness [78]. It is possible that the predominance of the anaerobic glycolytic pathway of ATP synthesis in stem cells not only maintains their stemness but also increases their resistance to stress signals leading to mitochondrial damage and cell death. In cancer cells, where, as in MSCs, glycolytic metabolism predominates, it has been shown that glycolysis is one of the key factors in their resistance to apoptosis [79]. Glycolysis inhibition makes cancer cells susceptible to Fas-induced apoptosis [80]. Another possible mechanism that protects MSCs from cell death may be the transfer of mitochondria between the cells. Since not all cells in a population respond equally to apoptotic stimuli [66,71,81], some cells can retain intact mitochondria and, therefore, can exchange them with the cells containing damaged ones [82], thereby saving the latter from cell death [83]. We suggest that the resistance of human L-MSCs to Fas-induced cell death may be mediated by the above mechanisms, but these issues still require further study.

To date, it has been proven that various cell types become more sensitive to Fas-induced apoptosis when they are in an inflammatory environment. Thus, it is assumed that after transplantation, MSCs, being in inflammatory conditions, become sensitized to cell death signals [14,64]. Several studies have shown that pro-inflammatory cytokines such as IL-1β, IFN-γ, and TNF-α increase the sensitivity of muscle cells [84], trophoblastic cells [85], non-activated T cells [86], skin fibroblasts [70], and others to Fas-induced apoptosis in vitro. This sensitization occurs due to an increased expression of caspases 8 and 3 [70,85] and/or an increase in Fas expression on the cell surface [70,84]. Other possible mechanisms are a decreased expression of the anti-apoptotic FLIP or an increased expression of the pro-apoptotic molecule BAX [70,86]. In our work, we showed that even after the treatment of human L-MSCs with the pro-inflammatory cytokine TNF-α, the cells did not become sensitive to Fas-induced cell death. It is still not entirely clear, what is the reason for such an increased resistance of L-MSCs to receptor-mediated apoptosis, and further detailed studies are required.

## 5. Conclusions

Our results indicate that human L-MSCs have an increased resistance to Fas receptor-mediated cell death both in normal and under inflammatory conditions. Whether this fact is a unique feature of L-MSCs or is also characteristic of MSCs obtained from other tissues remains to be established. Due to the lack of knowledge on the L-MSC fate after transplantation into animals, it remains unclear whether apoptosis of these cells occurs in the recipient’s body in vivo. If so, what are the signals and downstream mechanisms that trigger it? Do L-MSCs need any additional stimuli to undergo apoptosis in vivo? And is the potential to form apoptotic bodies in the case of L-MSCs a therapeutically significant process? All these questions remain to be answered in order to make it possible to assess the physiological role and significance of apoptotic cell death for the realization of the therapeutic potential of MSCs.

## Figures and Tables

**Table 1:** Percentages (mean ± SEM) of L-MSCs with a decreased mitochondrial membrane potential.

	Liver MSC#1	Liver MSC#2	Liver MSC#3
Control	5.2 ± 0.9	4.41 ± 0.8	7.3 ± 2.8
CoCl_2_, 400 µM	21.4 ± 0.9	19.3 ± 0.9	22.5 ± 3.2
Anti-Fas mAb, 2 µg/mL	46.2 ± 4.8	40.4 ± 2.5	48.9 ± 6.0
FasL, 500 ng/mL	51.7 ± 4.2	56.2 ± 5.1	47.5 ± 2.5
STS, 100 nM	24.4 ± 3.2	20.2 ± 1.9	26.7 ± 5.9
STS, 500 nM	48.3 ± 4.0	43.1 ± 3.6	52.4 ± 5.5

## Supplementary Materials

The following supporting information can be downloaded at: https://www.mdpi.com/article/10.3390/cimb44080236/s1, Figure S1: Mesodermal lineage differentiation of human L-MSCs; Figure S2: Flow cytometry analysis of L-MSC cell death after treatment with anti-Fas mAb or FasL. The cells were incubated with inductors for 24 h; Figure S3: Flow cytometry analysis of L-MSC cell death after treatment with anti-Fas mAb or FasL. The cells were incubated with inductors for 48 h; Figure S4. Flow cytometry analysis of L-MSC cell death after treatment with anti-Fas mAb or FasL. The cells were incubated with inductors for 72 h; Figure S5. L-MSC viability was analyzed by MTT-assay after treatment with different concentrations of FasL (A), anti-Fas mAb (B), CoCl2 (C) or STS (D) for 72 h; Figure S6. Flow cytometry analysis of mitochondrial membrane potential using JC-1 dye. L-MSCs were incubated with low concentrations of anti-Fas mAb or FasL (50 and 100 ng/mL) for 4 h; Figure S7. L-MSC (L-MSC#3) viability was analyzed by MTT-assay after treatment with different concentrations of cytokines for 48 h.
