# Supplementary material for: Resistance of Human Liver Mesenchymal Stem Cells to FAS-Induced Cell Death
Source: Curr Issues Mol Biol. 2022 Jul 30;44(8):3428–43. doi: 10.3390/cimb44080236 (PMC9406952; doi:10.3390/cimb44080236)
Supplement: Supplementary file 1 [file cimb-44-00236-s001.zip › cimb-1797767-supplementary.pdf]

Supplementary files

Supplementary methods

Mesodermal lineage differentiation.

Adipogenic differentiation medium: DMEM/F12, supplemented with 10% FBS, 100 U/mL penicillin and 100 μg/mL streptomycin (all of them Gibco, Waltham, MA USA), 0.1 μM dexamethasone, 0.45 mM IBMX, 1μg/mL insulin, 0.2 mM indomethacin (all of them Merck Life Science LLC, Moscow, Russia). The medium was changed every three days. Cells were cultured for 21 days. At the end of differentiation, cells were stained with Oil Red O (Merck Life Science LLC, Moscow, Russia).

Osteogenic differentiation medium: DMEM/F12, supplemented with 10% FBS, 100 U/mL penicillin and 100 μg/mL streptomycin (all of them Gibco, Waltham, MA USA), 0.1 μM dexamethasone, 10 mM β-glycerophosphate, 1μg/mL insulin, 250 μM ascorbic acid (all of them Merck Life Science LLC, Moscow, Russia). The medium was changed every three days. Cells were cultured for 21 days. At the end of differentiation, cells were stained with Alizarin Red (Merck Life Science LLC, Moscow, Russia).

MTT-assay for cell viability analysis.

A colorimetric MTT test was used to assess cell viability of human L-MSCs after treatment with FasL or anti-Fas mAb. The inductors were diluted in wells of a flat-bottom 96-well plate in a concentration range for FasL from 3-200 ng/mL and for anti-Fas mAb from 0.03-2 μg/mL (dilution step 1/2, 7 dilutions), after which cells were added to the wells (20,000 /well) and cultured for 72 h in a standard culture conditions. As a positive control for the metabolic activity recorded by MTT assay L-MSCs were incubated with various dilutions of $CoCl_2$ and STS for 72 h. In addition, the effect of cytokines, such as TNFα, IFNγ, TGFβ, and IL6, on the viability of MSCs was also evaluated by MTT assay. Each point was reproduced in three replications. L-MSCs cultured under the same conditions in the absence of inductors served as the control. After culturing, the medium was carefully removed from the wells; 30 μl MTT (Merck Life Science LLC, Moscow, Russia) was added to a final concentration of 0.5 mg/mL, and incubated for at least 2 h in a CO_2 incubator until the formation of formazan crystals. Then, DMSO (PanEco, Moscow, Russia) was added to the wells to dissolve the formazan crystals (100 μl/well). Optical density (OD) was measured using a Tecan infinite M200 Pro plate reader (Tecan, Männedorf, Switzerland) at 565 nm.

Cell viability was calculated by the formula:

(ODind. cells-ODblank)/(ODcontr. cells-ODblank)×100%,

where ODind. cells is optical density in wells containing cells treated with inducers or cytokines, ODblank is optical density in wells containing MTT and DMSO, but not containing cells. OD of control cells (ODcontr. cells) is optical density in wells containing intact cells.

Supplementary data

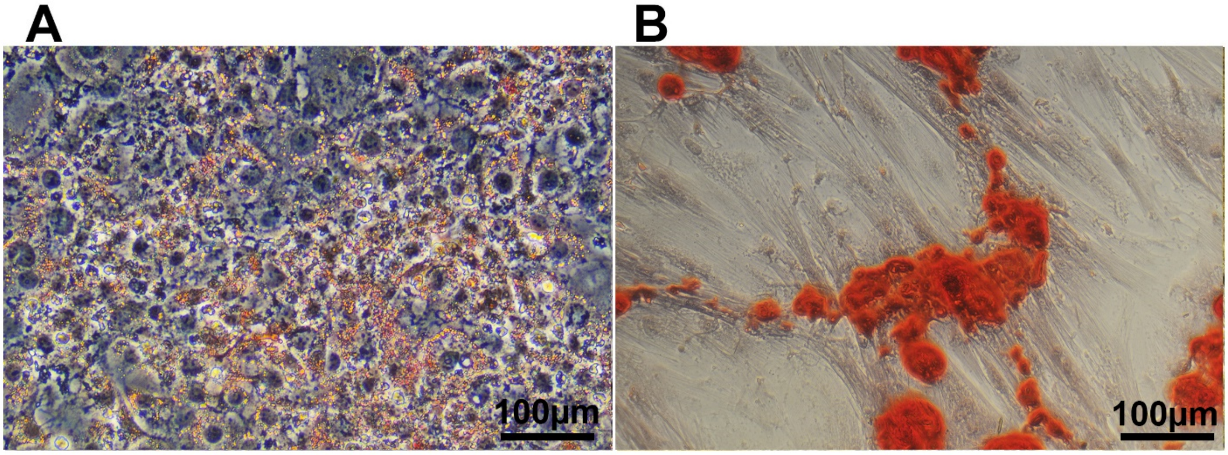

Figure S1. Mesodermal lineage differentiation of human L-MSCs. (**A**) Oil Red O staining after adipogenic differentiation of L-MSCs at 21 days. (**B**) Alizarin Red staining after osteogenic differentiation of L-MSCs at 21 days. Photographs were prepared by using the Axiovert 40 CFL (Carl Zeiss, Oberkochen, Germany) inverted microscope and the Nikon D5000 digital camera (Nikon, Tokyo, Japan).

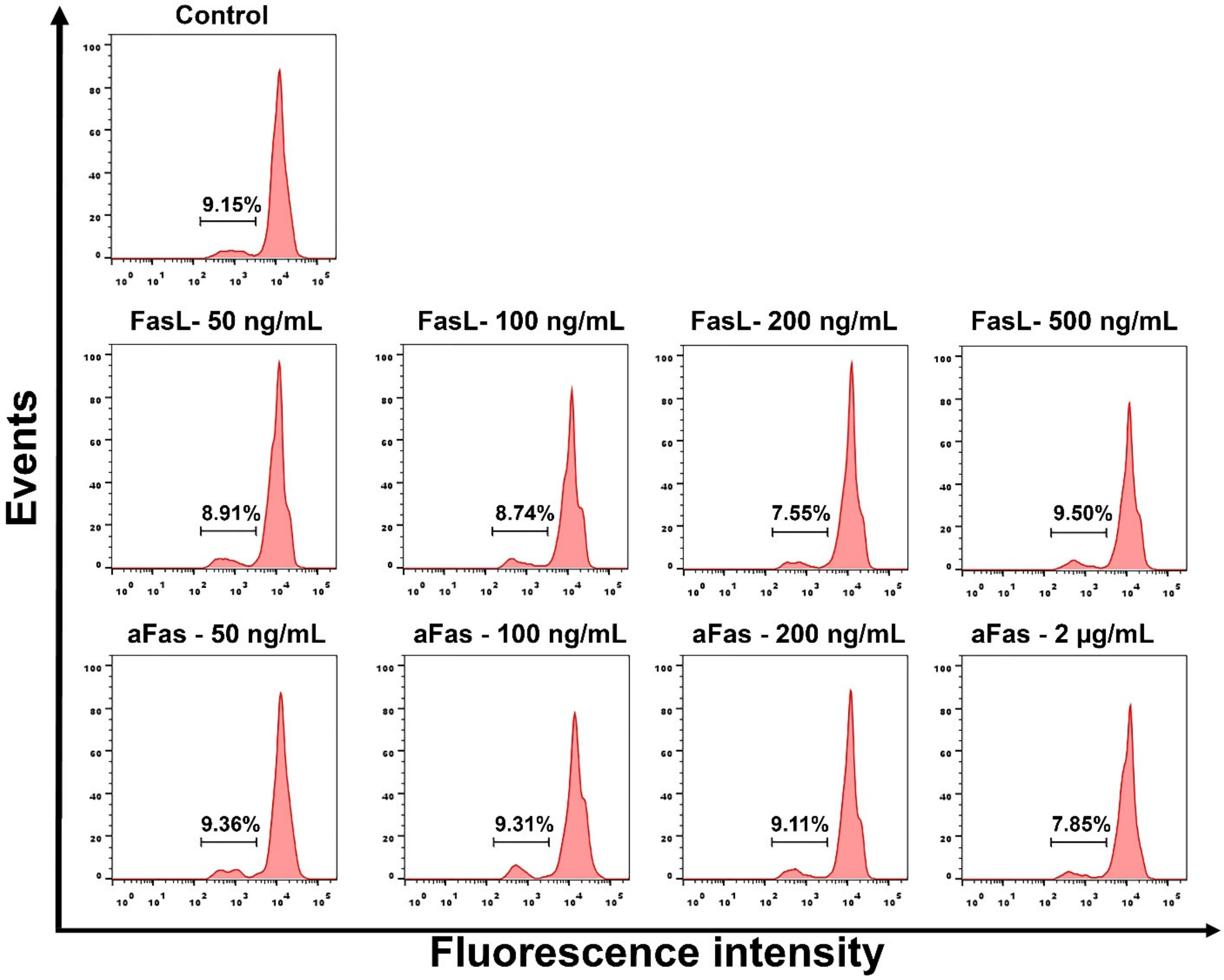

Figure S2. Flow cytometry analysis of L-MSC cell death after treatment with anti-Fas mAb or FasL. The cells were incubated with inductors for 24h; and then cells were fixed with ice cold ethanol and stained with PI. The figure shows representative data for one culture of L-MSCs (L-MSC#2). The marker shows the number of cells (%) with fragmented DNA.

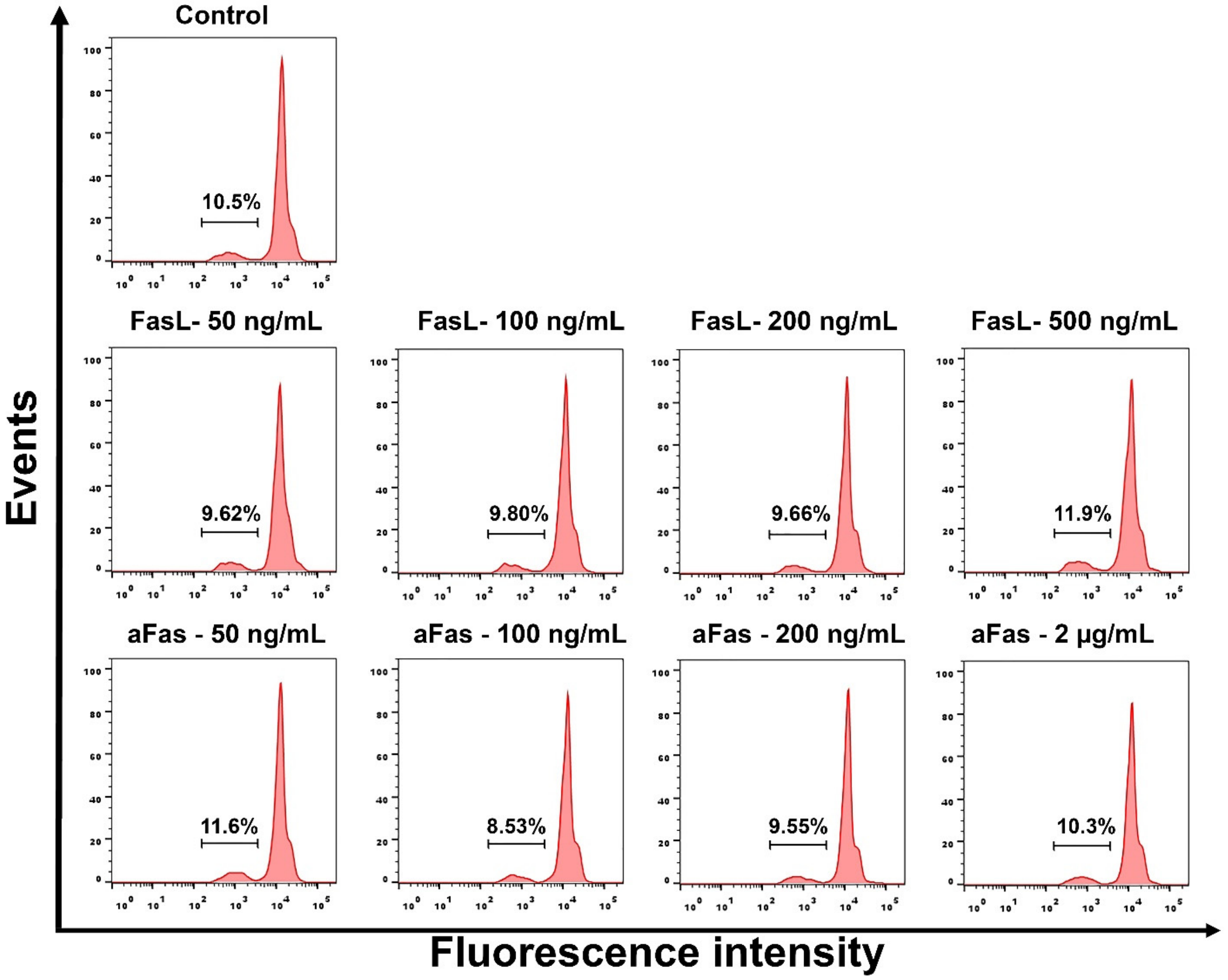

Figure S3. Flow cytometry analysis of L-MSC cell death after treatment with anti-Fas mAb or FasL. The cells were incubated with inductors for 48h; and then cells were fixed with ice cold ethanol and stained with PI. The figure shows representative data for one culture of L-MSCs (L-MSC#2). The marker shows the number of cells (%) with fragmented DNA.

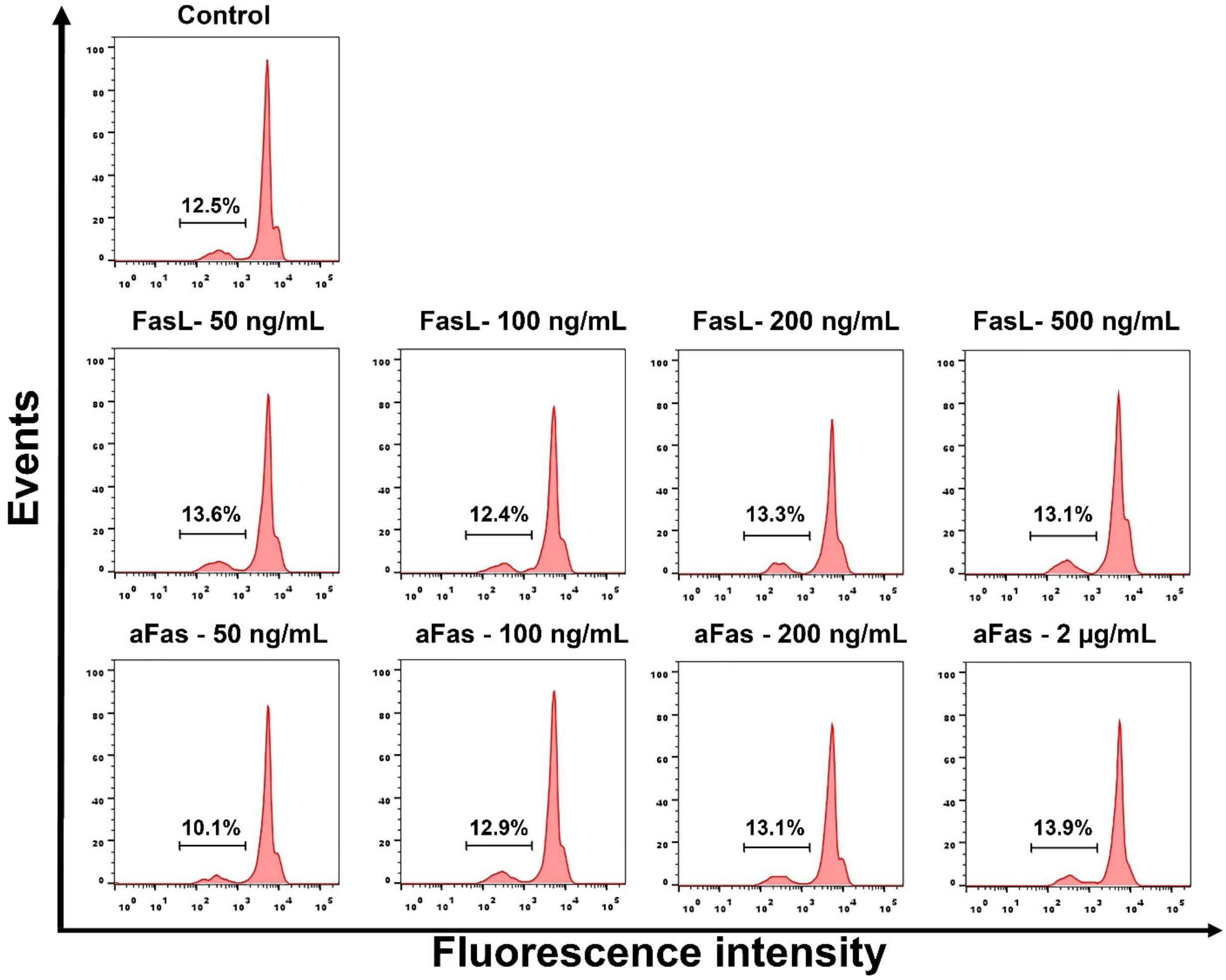

Figure S4. Flow cytometry analysis of L-MSC cell death after treatment with anti-Fas mAb or FasL. The cells were incubated with inductors for 72h; and then cells were fixed with ice cold ethanol and stained with PI. The figure shows representative data for one culture of L-MSCs (L-MSC#2). The marker shows the number of cells (%) with fragmented DNA.

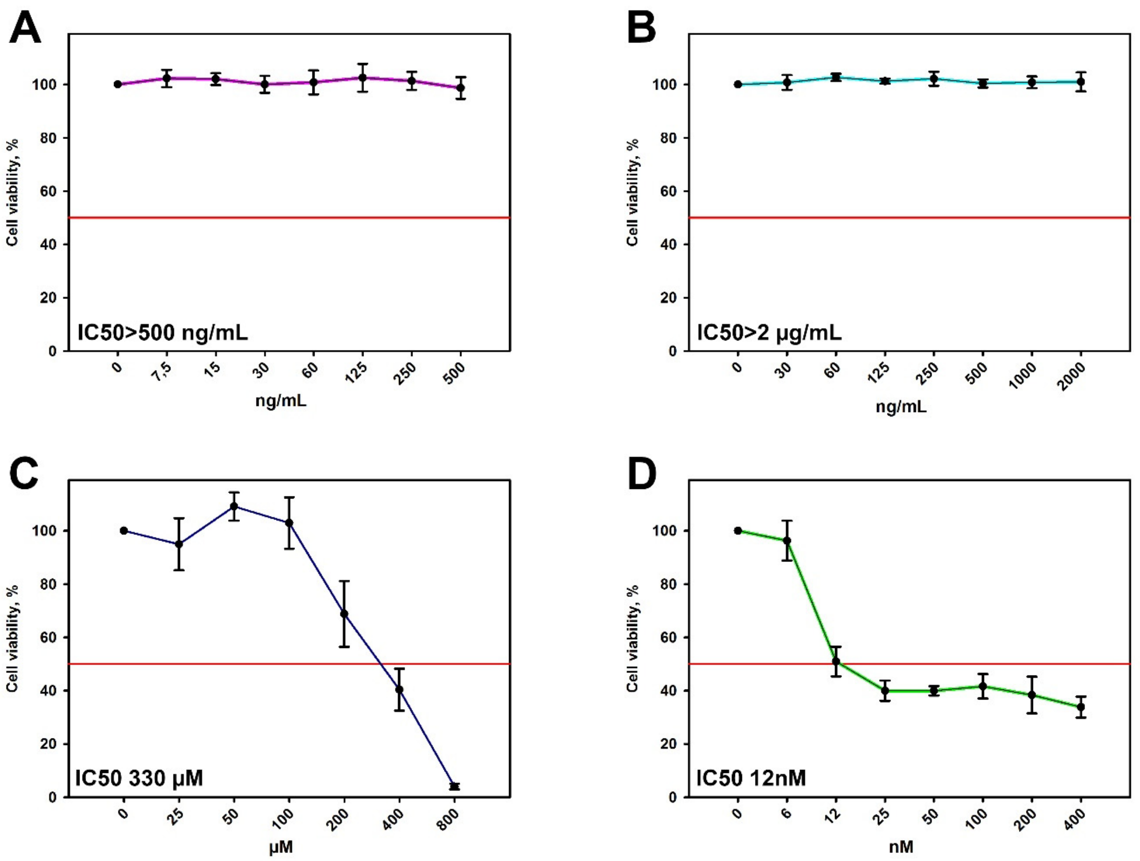

Figure S5. L-MSC viability was analyzed by MTT-assay after treatment with different concentrations of FasL (**A**), anti-Fas mAb (**B**), CoCl2 (**C**) or STS (**D**) for 72h.

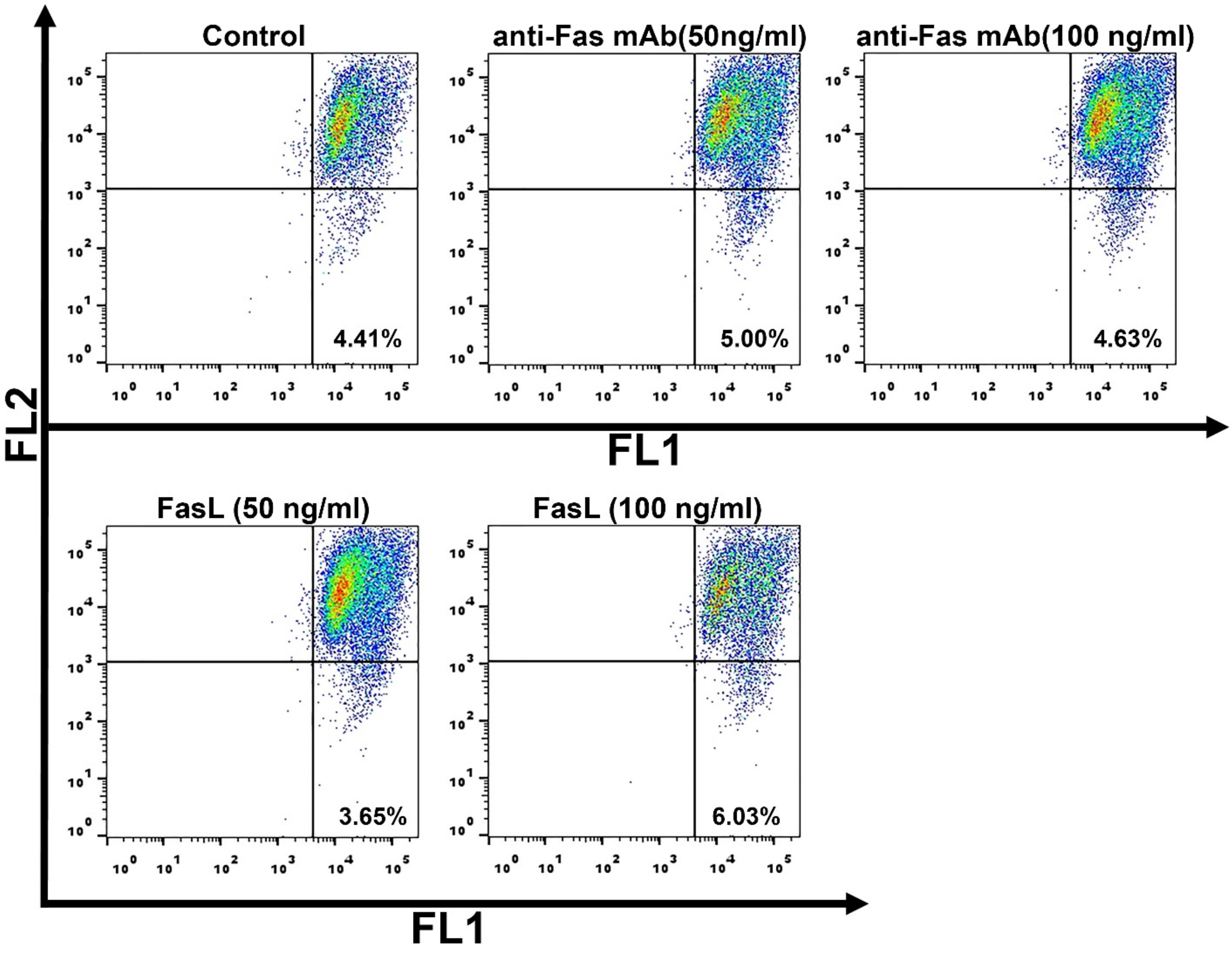

Figure S6. Flow cytometry analysis of mitochondrial membrane potential using JC-1 dye. L-MSCs were incubated with low concentrations of anti-Fas mAb or FasL (50 and 100 ng/mL) for 4 h. Representative data for a single culture of L-MSCs are shown.

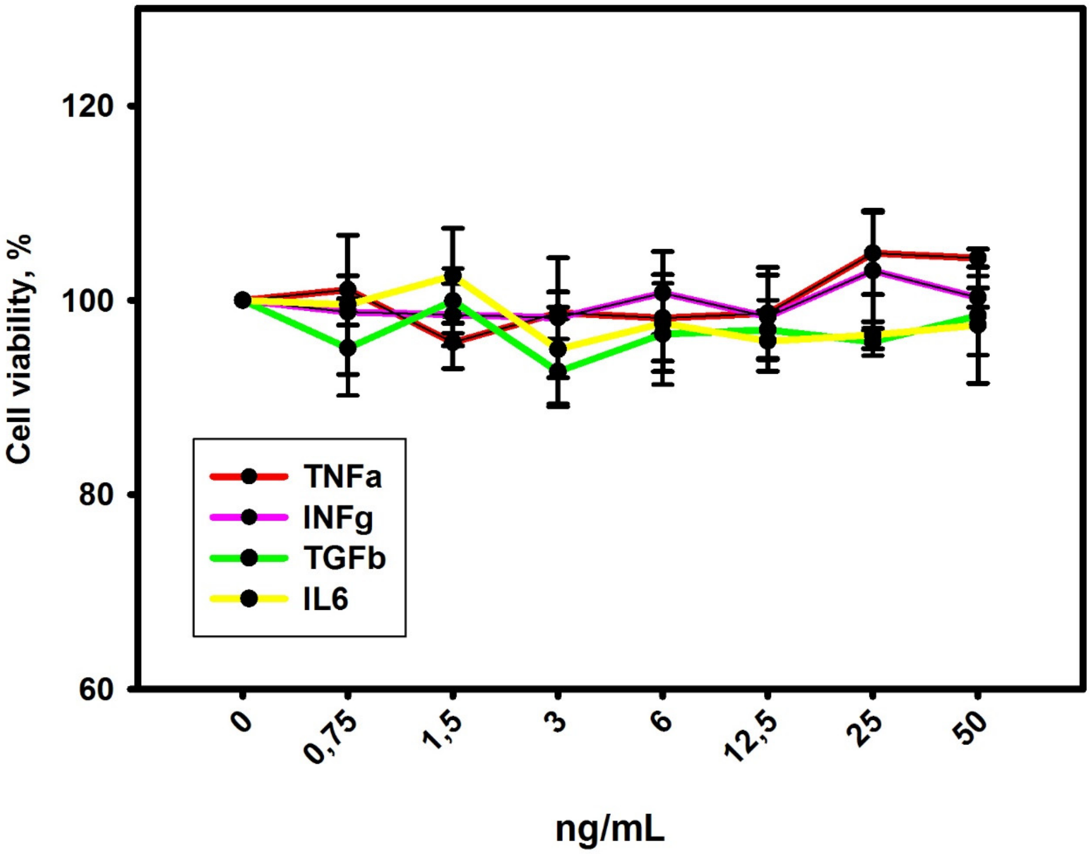

Figure S7. L-MSC (L-MSC#3) viability was analyzed by MTT-assay after treatment with different concentrations of cytokines for 48 h.
